# Do Physical Proximity and Availability of Adequate Infrastructure at Public Health Facility Increase Institutional Delivery? A Three Level Hierarchical Model Approach

**DOI:** 10.1371/journal.pone.0144352

**Published:** 2015-12-21

**Authors:** Rachana Patel, Laishram Ladusingh

**Affiliations:** 1 International Institute for population Sciences (IIPS), Mumbai, India; 2 Department of Mathematical Demography and Statistics, International Institute for population Sciences (IIPS), Mumbai, India; National Cancer Institute, UNITED STATES

## Abstract

This study aims to examine the inter-district and inter-village variation of utilization of health services for institutional births in EAG states in presence of rural health program and availability of infrastructures. District Level Household Survey-III (2007–08) data on delivery care and facility information was used for the purpose. Bivariate results examined the utilization pattern by states in presence of correlates of women related while a three-level hierarchical multilevel model illustrates the effect of accessibility, availability of health facility and community health program variables on the utilization of health services for institutional births. The study found a satisfactory improvement in state Rajasthan, Madhya Pradesh and Orissa, importantly, in Bihar and Uttaranchal. The study showed that increasing distance from health facility discouraged institutional births and there was a rapid decline of more than 50% for institutional delivery as the distance to public health facility exceeded 10 km. Additionally, skilled female health worker (ANM) and observed improved public health facility led to significantly increase the probability of utilization as compared to non-skilled ANM and not-improved health centers. Adequacy of essential equipment/laboratory services required for maternal care significantly encouraged deliveries at public health facility. District/village variables neighborhood poverty was negatively related to institutional delivery while higher education levels in the village and women’s residing in more urbanized districts increased the utilization. “Inter-district” variation was 14 percent whereas “between-villages” variation for the utilization was 11 percent variation once controlled for all the three-level variables in the model. This study suggests that the mere availability of health facilities is necessary but not sufficient condition to promote utilization until the quality of service is inadequate and inaccessible considering the inter-districts variation for the program implementation.

## Introduction

India continues to contribute about a quarter of all global maternal deaths; however, it experienced declined in Maternal Mortality Ratio (MMR) from 301 to 254 per 100,000 live births during 2001–2006 [1–2]. A study from under-developed country showed that 96 per cent of pregnant women had at least one antenatal check-up and that only half delivered in a health facility, the assessment found substantial gaps in the availability and quality of care [3]. Appropriate delivery care is crucial for both maternal and perinatal health and increasing skilled attendance at birth is a central goal of the safe motherhood and child survival [4]. Since, it is established that maternal deaths are closely associated with the institutional birth and that’s why every policy focusing to reduce maternal deaths has aim to encourage the institutional births (preferably) or delivery by skilled births attendant. The Indian national health policy (2000) envisages 100% institutional delivery.

Undoubtedly, socio-economic and demographic factors are stronger predictors of health care utilization than the accessibility of health services [5–7]. But a number of studies have found high utilization of health services whenever a health facility is present in the community or neighborhood. In Guatemala, the availability of private physicians and government-sponsored health services within communities had only a modest effect, relative to the effects of socio-economic factors, on rural women's decisions to obtain health care during pregnancy [8]. As regards the community effect on health facility utilization, earlier studies have found that people living in the poorest neighborhoods are least likely to receive adequate care [9–11]. As Stephenson and Tsui [12] remarked that individual and household characteristics and have largely ignored the influence of community attributes and the characteristics of the health services available on the use of reproductive health-care services. In Chiapas, for example, intra-community division of political affiliation is associated with more home deliveries and in Uttar Pradesh, women in more populous communities are less likely to deliver in a facility [13].

Studies on determinants of maternal health care utilization pay little attention to availability and accessibility of health facilities, though these factors are on par with socio-demographic and economic factors. This is partly due to the lack of adequate data on health facility. Recently there has been an increasing interest in the ways in which health services influence care-seeking behavior. In the 'three-delays' model [14], for instance, Thaddeus and Maine (1994) [15] emphasize how community differences in access to health facilities, the availability of healthcare providers, and the adequacy of transport systems may influence timely care-seeking for obstetric complications and, ultimately, maternal mortality in less developed countries. Distance is a crucial dimension of the utilization of health services, yet its relevance to women's decisions to seek pregnancy and delivery care has not been well explored. Research has shown that in Uganda, access to maternity services was an important determinant of the choice of delivery site [16]. Stephenson and Tsui [13] have provided evidence that in Uttar Pradesh, the number of doctors in the community significantly promotes utilization of health facilities for delivery-care services, while the presence of a secondary health facility considerably enhances care-seeking for both pregnancy and childbirth.

**Fig 1:**
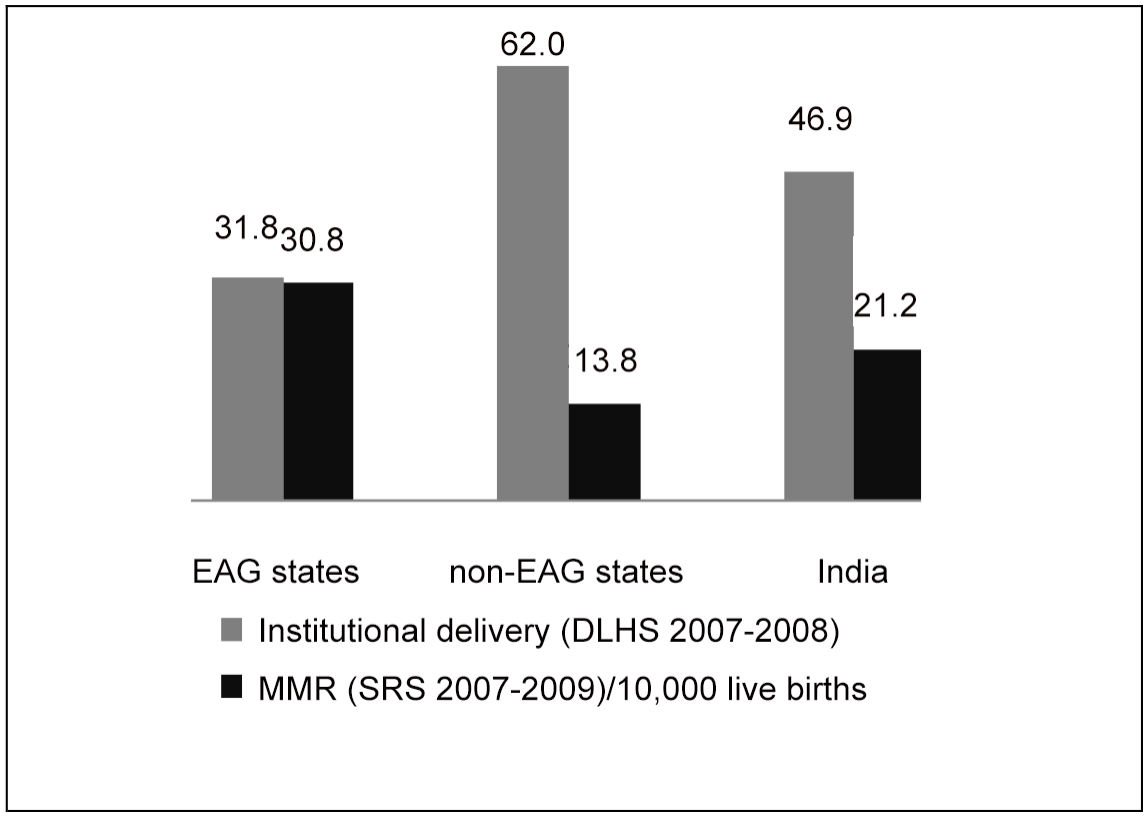
Institutional Births (%) and MMR (per 10,000 live births) in EAG and non-EAG states India, 2007–08. MMR refers to Maternal Mortality Ratio. Institutional birth refers the birth delivery in any type of health care center (private/public). EAG states are Bihar, Jharkhand, Uttar Pradesh, Uttaranchal, Rajasthan, Orissa, Madhya Pradesh and Chhattisgarh. Since DLHS data gives only institutional delivery information hence SRS data was used for MMR. Similar reference time was kept in both data.

**Fig 2:**
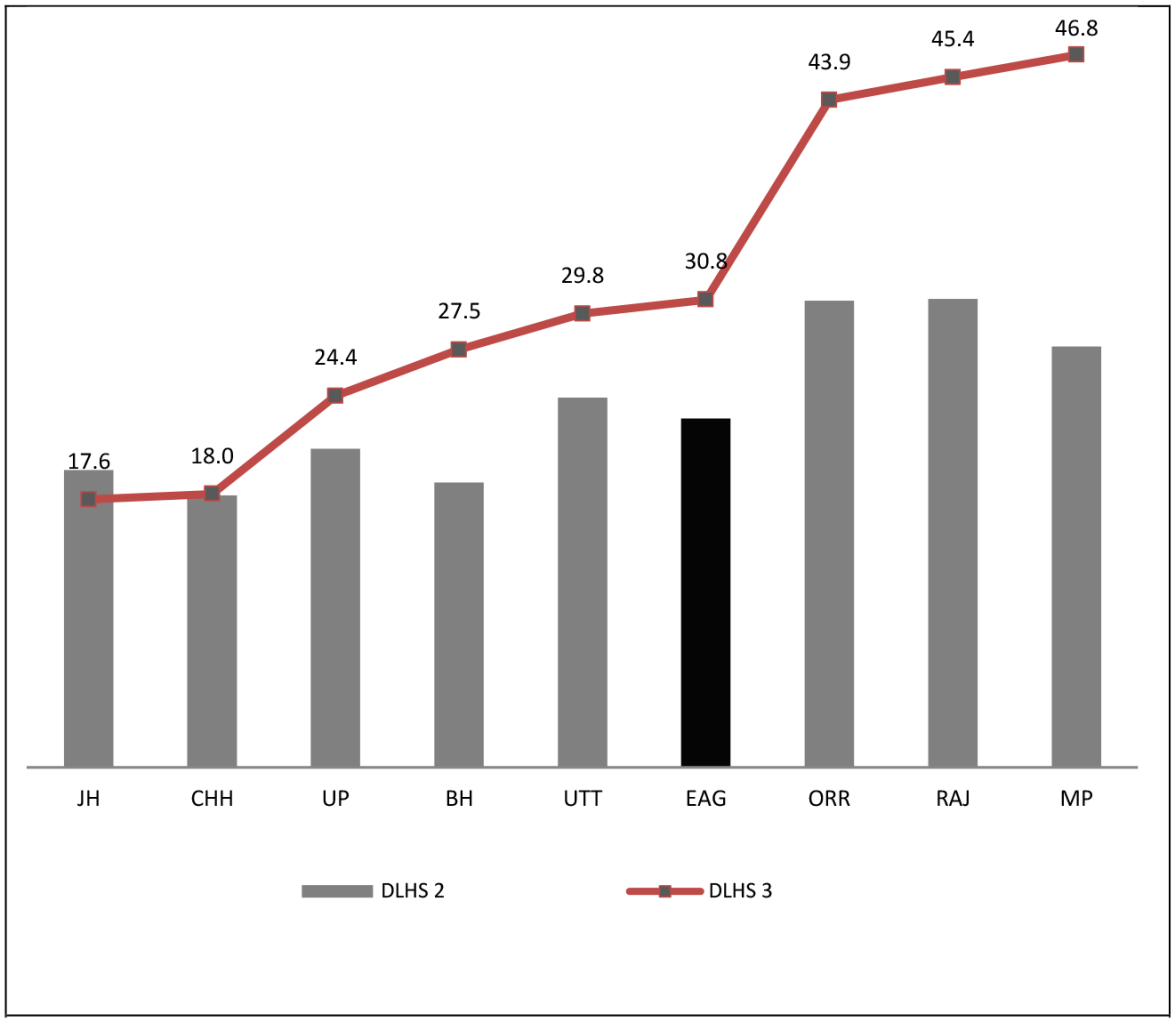
Institutional Births (%) in EAG states, DLHS-2(2002–2004) and DLHS-3 (2007–2008). EAG value is average of all 8 states.

About every second women have an institutional delivery, three anti-natal visits (ANC) and receiving folic acid for at least 100 days during pregnancy [17]. Staggered economy and huge population demand have had great repercussions on India's health system. With the exception of few southern regions [18], and a few urban areas, there is a marked shortage of equipment and qualified personnel for meeting maternal care needs. Apparently, utilization of health facilities for institutional delivery in most of the northern states of India is far from satisfactorily in general and particularly in the EAG states compared to southern states. The EAG states of Bihar, Jharkhand, Uttar Pradesh, Uttaranchal, Rajasthan, Orissa, Madhya Pradesh and Chhattisgarh constitute more than 45 percent of the India’s population. The negative association between institutional births and MMR was found to be (Fig 1) very low 31 percent institutional births and very high MMR in EAG states compared to non-EAG and it is even below the national average. However, during DLHS-II [19] and DLHS-III [20] the increase in institutional delivery could be seen in some of the states of EAG region but few states are still lacking in the level of improvement (Fig 2). Partly this is due to uneven in distribution of facilities, inadequate supplies, insufficient effective person hours, unbalanced distribution of time to essential activities. Absence of effective available services, affordable, acceptable and quality health facilities may seriously hinder utilization for maternal care [21–22].

State programs were decentralized and National Rural Health Mission (NRHM, 2005) was launched to meet the challenges for ensuring maternal and child health services, under which Health Sub-Centre (HSC) and Primary Health Centre (PHC) were strengthened and; training of village health volunteers and institutional deliveries were promoted at grass root level (village/block) for improving health care delivery across rural India under the auspices of the Ministry of Health and Family Welfare (MOHFW). The NRHM introduced new incentive schemes for maternal healthcare delivery including a local health worker (Auxiliary Nurse Midwife -ANM/ Accredited Social Health Activist-ASHA), resident of same locality, who assists in bringing women for institutional delivery and the Janani Suraksha Yojana (JSY) which provides cash compensation to women for institutional delivery. This aims to provide effective maternal healthcare to the rural population, especially the vulnerable sections throughout the country with special focus on 18 states, which have weak public health indicators and/or weak infrastructure (NRHM, 2011).

To monitor the effectiveness of provision of maternal and child health care in the Indian health system, this study attempts to explore the association between accessibility and adequacy of health facilities with maternal care in rural northern India. Maternal care outcome institutional birth is a part of the key goals of the government safe motherhood program (JSY) that reflects WHO recommendations for the detection to improve maternal and child health complications to reduce maternal mortality [23].

## Conceptual Framework

Consequently, instead of limiting the conceptual framework suggested by Andersen and Newman model [24], a modified framework with its attendant covariates was adopted in this study. Few health facility and accessibility variables are included as environmental factors to modify the framework accordingly. In addition to predisposing, enabling and need factors (delivery complications and earlier experienced any pregnancy loss), some external correlates are connectivity, availability and distance to facility. Importantly, facility and health program factors like availability of health centers, adequate manpower and infrastructure, functional facilities available, maternal health care program in village/community are considered as environmental factors and the data structure are given in S1 Fig.

### Health facility adequacy indices

Some studies conducted in the recent years have attempted to develop multi-dimensional scales and measure the quality of healthcare services in the developing nations. Haddad, Fournier and Potvin [25], developed and validated a 20-item instrument for health utilization in Guinea for the dimension of healthcare delivery, personnel, and health facility. Later, Baltussen et al [26] adapted this scale in the context of Burkina Faso and identified four dimensions of healthcare quality. Duong et al. [27] have also demonstrated the feasibility, reliability, and validity of the instrument developed by Haddad, Fournier and Potvin [25] in the context of rural Vietnam. They identified four factors to measure the client perceived quality: healthcare delivery, health facility, interpersonal aspects of care, and access to services.

Similar to that, this study’s first aim was to assess the adequacy of infrastructure required for maternal care, available at HSC and PHC using the facility survey of DLHS-3. It is important to scrutinize the adequacy of these facilities, as HSCs and PHCs are setup in rural areas to facilitate the decentralized government health program (NRHM, 2005) and to meet the maternal health care needs at the grass-root level. Through the assistance of expert gynecologist, the study listed the essential equipment/instruments, manpower and drugs required for delivery, availability of components were coded as 1 and 0, for the construction of adequacy indices.

### Primary Health Center (PHC) level indices

At the village level, the resources of the PHCs should be able to adequately provide all services related to maternal and child health care including the three major components of pre-natal, delivery and post natal care to the pregnant women. Indices relating to functioning of PHCs are outlined below:

1. **Manpower index:** Availability of trained health professionals at the health center is the primary requirement for institutional birth delivery. A study by **López-Cevallos D F et.al.** [28] shows that the density of public health practitioners was positively associated with health care utilization in rural areas. Here composite index for manpower required for delivery at health facility included 8 essential personnel for institutional delivery.
2. **Physical infrastructure index:** Adequate physical infrastructure is crucial to create an incentive for women to have an institutional delivery and consequently to improve MCH care utilization. In context to its implications for maternal care, this index included a total of 10 items.
3. **Essential drugs index:** This index comprised of 13 essential drugs including anti-allergics and drugs used in anaphylaxis, anti-hypertensive, anti-diabetics, solutions correcting water/electrolyte imbalance and essential obstetric care drugs.
4. **Essential delivery care equipment index:** This index included a total of 39 instruments/lab services, including kits, cold storage devices and so on, which are essential for conducting a delivery in a health facility.

### Health Sub-Center (HSC) level indices

HSC is the primary health set-up in a village, which aims to provide basic health care to the pregnant women and their child. Each HSC employs an ANM and a community ASHA worker, who are supposed to create awareness and provide information to the community on determinants of health. The following were the indices constructed to assess facilities in HSCs.

1. **Physical infrastructure index:** This index included a total of 35 variables around the availability of instruments and of adequate essential drugs relevant to maternal health care, as recommended by the NRHM. This index also covered manpower included the availability of a skilled ANM (trained attendant).

## Materials and Methods

### Data Source

This study uses household and facility data from the District Level Health Survey (DLHS-III, 2007–08), which was a nationally representative survey of households and health facility at district level. The study adopted a two-stage stratified sampling in rural areas. The data collection comprised of two surveys covering households and health facilities. Semi-structured questionnaires were designed to collect information on the households, including ever married women, unmarried women, and on the village. In the survey on health facilities, data about manpower, medicines, equipment and infrastructure for all levels of health facilities was collected. These constitute the Health Sub-Centre (HSC), Primary Health Centre (PHC), Community Health Centre (CHC) and District Hospitals (DH).

For the last child born in the 5 years preceding the survey to women in ages 15–49, data was collected regarding ANC source, content and frequency, the timing of the first visit, status of tetanus toxoid vaccination (TT), and place of delivery and the professional who conducted the delivery. The village information included road conditions, distance to the nearest health center, mode of transport to the service center, and existent health services (health centre, doctor, health worker, midwife/birth attendant, etc.). Each public health facility collected information on services provided (ANC, delivery care, post-delivery care, vaccinations, curative care) and the infrastructure available there. Further details of the survey design can be obtained from the country report [19].

This study was restricted to rural areas of eight socio-economically under developed states in north India which collectively share 40 percent of the country’s population. These included: Uttar Pradesh, Uttaranchal, Bihar, Jharkhand, Madhya Pradesh, Chhattisgarh, Orissa and Rajasthan. The analysis was based on 55043 births in the five years period preceding the survey, to rural women from 5687 villages from 263 districts of these states.

### Variables description

Based on the Andersen’s framework of health service utilization, variables from the three-level model were identified as predisposing, enabling, need and environmental factors. The following are the variables chosen at each level MLwin 2.11 version was used to get results from multilevel modeling.

1. **Individual-level**: Children’s Mother's socio-demographic characteristics like level of education; age at birth; caste, childcare burden; working status; partner’s education; information received on ANC/institutional birth; relative socio-economic status (household wealth quintile), JSY received, at least 3 ANC received; and child birth order. While delivery complications and any pregnancy loss in last five years are considered as need factors. All individual-level variables were coded categorically; additionally mode of transport and household wealth quintiles is included.
2. **Neighborhood-level (villages or PSU) variables**:In this study primary sampling units (PSUs) which are sampled villages are considered as neighborhood and that could be divided into two parts, one is accessibility and availability of health center from village; and other is related to health program variables in village or near to village. Accessibility and availability variables are all weather road connectivity to health center and distance to nearest public hospital. Program variables are: Concentration of population educated to secondary or higher level, ANM availability and skilled health attendant facilitating ANC available in village, improved status of HSC/PHC/CHC health facility adequacy indices (physical infrastructure, health personnel, essential drugs and equipments, instruments at PHC and HSC level). All variables are coded categorically.
3. **District level variable:** Few variables are chosen at district level which may have more influence on outcome variable like percent urban population, percent proportion of households belong to lowest wealth quintile (household assets based) and average number of delivery at HSC and PHC. MLWin 2.11 version was used to get results from multilevel modeling.

### Statistical Analysis

#### Reliability test of adequacy Indices and Principle Component Analysis (PCA)

Reliability of the health facility adequacy indices, discussed in the preceding section are tested by Cronbach’s alpha. Its value ranged between 0 and 1. The closer the coefficient alpha is to 1.0 the greater is the internal consistency of the items included in the index. Both, the number of items in the scale and the mean inter-item correlations determine the size of alpha. The Cronbach’s alpha values of the indices are shown in Table 1. The range of Cronbach’s alpha values for PHC based indices is from 0.66 to 0.96 and value based on HSC is 0.84 therefore by thumb rule they are acceptable for index preparation. The reliability was highest for the index of adequacy of essential equipments/laboratory services availability for delivery care’ (0.96) and lowest for the index of manpower adequacy (0.66) at PHC.

**Table 1:** Summary statistics and degree of reliability for adequacy indices.

	Indices of health facility adequacy	min	max	mean	SD	Average inter-item covariance	alpha
**Public Health Centre (PHC)**							
**1**	Manpower index	0	8	4.4	2	0.0408	0.6615
**2**	Physical infrastructure index	0	10	6.9	1.8	0.0365	0.7196
**3**	Essential drug index	0	13	7.6	3.5	0.0579	0.8265
**4**	Essential equipment/ Laboratory services index	0	39	18.5	4.6	0.0517	0.9593
**Health Sub-Centre (HSC)**							
**1**	Physical infrastructure index*	0	3	22	2	0.041	0.84

* includes manpower ANM (other health worker), physical infrastructure, drugs availability, equipment at HSC.

Principle component analysis (PCA through STATA 10) was used to examine the structure of the relationship among items included in the construction of the above health facility adequacy indices. The generated score of Kaiser–Meyer–Olkin (KMO) was 0.82 and test of sphericity by Bartlett’s is highly significant supporting the appropriateness of using factor analysis to explore the underlying structure of perceived quality of healthcare services. An “eigen value greater than 1” criterion was employed for determining the number of factors. Factor loadings of 0.5 or greater on a factor were regarded as significant. PCA scores were obtained from the selected variables at health facility for each index and quintiles were created separately for those indices.

#### Bivariate and multivariate model

The essential indicators of maternal care that this study focused on institutional delivery and included three ante natal care (ANC) however multilevel modeling is done for institutional delivery only as outcome. Bivariate analysis was carried out with outcome variable by EAG states before processing the multilevel modeling. Correlation matrix for the selected program and district level variable was used to explain the choice of variables for regression model.

The study used multilevel logistic regression to model delivery care outcome adjusting for district/ neighborhood effects and socio-demographic background of mothers. Multilevel model accounts hierarchical structure of the data included by clustering of births to mothers within villages (primary sampling unit), and villages within districts [29]. A three level model considering births at level 1, villages at level 2 and districts at level 3 was implemented for multivariate analysis using MLwin version 2.11 [30]. The following is the form of the multilevel logit model used in the analysis:

$$logit\left(\pi_{ijk}\right)=\text{log}\left(\frac{\pi_{ijk}}{1-\pi_{ijk}}\right)=\beta_{0jk}+\;\beta_{ijk}I_{ijk}+\beta_{jk}P_{jk}+\;\beta_{k}D_{k}+\;\varepsilon_{ijk}$$ (1)

And

$$\beta_{0jk}=\beta_{0}+\upsilon_{0k}+\;u_{0jk}$$ (2)

where i, j and k indicates the levels 1, 2 and 3 respectively; *π*_*ijk*_ is the probability of uptake of maternal care of interest for the *i*th birth, in the *j*th village of *k*th district; and error term *ε*_ijk_ is assumed to follow normal distribution. Further I, P, and D are the vectors of mother (individual), village (PSU) and district level covariates respectively. υ_0k_ and u_0jk_ are random intercepts of “between district” and “between villages” variance respectively that is proportion of variation explained by district of level 3 and village of level 2 in total variation, which follow a normal distribution with mean zero and their covariance matrix for three-level model.

Two versions of multilevel model to examine the effect and significance of the individual, village and district level factors on the maternal care outcome are considered. In each model, the neighborhood-level random intercept represented the extent to which outcomes varied between neighborhoods after adjusting for confounders at different levels; it also represented other factors not considered in the model or those that could not be readily quantified in a large-scale survey, such as neighborhood variations in beliefs about delivery care.

## Results and Discussion

### Characteristics of the sample

Table 2 shows the descriptive statistics of district, village and individual levels correlates of institutional delivery included in the model. Neighborhood-level (village) variables capture the ability of potential users to reach health services physically. In districts of EAG states, on an average only 14 percent of the population belonged to urban and 28 percent of households belonged to the lowest wealth quintile. About a 7 percent population in the villages had obtained secondary or higher level education in the village. The program factors and their utilization showed that there was a shortage of skilled ANMs (30 percent). 65 percent of villages had a functional PHC, about 86 percent were connected by all- weather roads to the nearest health centre and 54 percent of villages demonstrated improvement in public health services in past few years; however only 5 percent improvement was stated as “very good”. Progressively, 50 percent PHCs showed more than 3/4^th^ health personnel availability and 73 percent PHCs were well equipped with essential drugs. On the other hand, only 33 percent of the PHCs and 37 percent HSCs were well equipped (upper 3^rd^ adequacy quintile) with essential equipment/instruments/laboratory services required for maternal care and physical infrastructure respectively.

**Table 2:** Unweighted summary statistics of variables used in modeling maternal health care (N = 55043).

Factors	Category/coding	Level	Mean	SE
**Predisposing factors**				
**Age at birth**	<25 years = 1	Individual	0.49	0.002
	25–29 years = 2		0.29	0.002
	30 & above years = 3		0.22	0.002
**Caste**	SC/ST = 1	Individual	0.37	0.002
	OBC = 2		0.48	0.002
	Others = 3		0.14	0.001
**Birth order**	One = 1	Individual	0.25	0.002
	2–3 = 2		0.4	0.002
	3 & more = 3		0.35	0.002
**Child care burden (additional child <5 years old)**	No another child = 0	Individual	0.03	0.001
	One another child = 1		0.71	0.002
	2+ children = 2		0.27	0.002
**Working women**	No = 0	Individual	0.51	0.002
	Yes = 1		0.49	0.002
**Enabling factors**				
**Education**	No education = 0	Individual	0.65	0.002
	<5 years = 1		0.07	0.001
	5–9 years = 2		0.21	0.002
	10 and above = 3		0.07	0.001
**Partner’s education**	No education = 0	Individual	0.34	0.002
	<5 years = 1		0.08	0.001
	5–9 years = 2		0.35	0.002
	10 and above = 3		0.23	0.002
**Information on institutional delivery**	No = 0	Individual	0.31	0.002
	Yes = 1		0.69	0.002
**JSY received**	No = 0	Individual	0.91	0.001
	Yes = 1		0.09	0.001
**3 and more ANC**	No = 0	Individual	0.73	0.002
	Yes = 1		0.27	0.002
**Wealth quintile**	Poorest = 1	Individual	0.36	0.002
	Second = 2		0.29	0.002
	Middle = 3		0.18	0.002
	Fourth = 4		0.12	0.001
	Richest = 5		0.05	0.001
**% household with higher education in village**	12th and above standard	Village/PSU	0.07	0.073
**% urban by district**		District	0.146	0.104
**% poorest household by district**		District	0.278	0.157
**Need factors**				
**Pregnancy loss in last 5 years**	No = 0	Individual	0.92	0.001
	Yes = 1		0.08	0.001
**Problem during delivery**	No = 0	Individual	0.29	0.002
	Yes = 1		0.71	0.002
**Environmental factors**				
**External environment factors**				
**Public health center accessible throughout the year**	No = 0	Village/PSU	0.14	0.002
	Yes = 1		0.86	0.002
**Private health center accessible throughout the year**	No = 0	Village/PSU	0.15	0.002
	Yes = 1		0.85	0.002
**Distance to public health center providing delivery care**	<10 km = 1	Village/PSU	0.68	0.002
	10-30km = 2		0.3	0.002
	30+ km = 3		0.02	0.001
**Community health program variables**				
**ANM in village (<5km)**	No = 0	Village/PSU	0.36	0.002
	Yes = 1		0.64	0.002
**Skilled ANM (skill attendant)**	No = 0	Village/PSU	0.7	0.002
	Yes = 1		0.3	0.002
**Functional PHC**	No = 0	Village/PSU	0.35	0.002
	Yes = 1		0.65	0.002
**Improved public health facility (SC/PHC/CHC)** #	Not good = 0	Village/PSU	0.4	0.002
	Good = 1		0.54	0.002
	Very good = 2		0.05	0.001
**Manpower adequacy at PHC**	<60% (3rd quintile) = 0	Village/PSU	0.5	0.002
	>60% (3rd quintile) = 1		0.5	0.002
**Drug adequacy at PHC**	<60% (3rd quintile) = 0	Village/PSU	0.27	0.002
	>60% (3rd quintile) = 1		0.73	0.002
**Equipment/lab services adequacy at PHC**	<60% (3rd quintile) = 0		0.67	0.002
	>60% (3rd quintile) = 1		0.33	0.002
**Infrastructure adequacy at HSC**	<60% (3rd quintile) = 1	Village/PSU	0.63	0.002
	>60% (3rd quintile) = 0		0.37	0.002
**Average number of delivery at SC/PHC by district**		District	37.6	52.4

^#^improved quality is very much subjective but here coding were done based on responses only.

Nearly 49 percent women were of age up to 25 years at the time of child-birth, 37 percent belonged to SC/ST castes followed by 48 percent from OBC castes. 40 percent women had birth of second order, and majority of women (about three in every four) had another child of age below five years already. 65 percent women were non-educated while their partner’s education, the figures are marginally better 34 percent were non-educated. Working and non-working women were equally distributed in the population. Further, the study considered some maternal health program- related information to see differentiation in the utilization pattern. The Government program on delivery care had successfully reached women either through media, health personnel or through some other source. About two third women had been informed about delivery care and about one in four had utilized at least 3ANC (as suggested under RCH the program for better maternal care). Only 10 percent women had benefited from JSY (incentives for institutional delivery).

### Bivariate results

Table 3 describes the percentage of delivery care of women and state-wise differential in prevalence of institutional delivery by accessibility and maternal care program variables. Test of difference shows the significant variation by program variables between states. States Uttaranchal, Rajasthan, Orissa and MP showed highest (more than 50 percent) institutional births while least progress in Jharkhand (29 percent) and Chhattisgarh (19 percent) to women who had gone for at least 3 ANC visits. More than 40 percent institutional deliveries occurred to women in same states followed by Bihar (35 percent) if they had received information about government program for the institutional delivery while dissimilar pattern for delivery care was observed by states in presence of JSY program in village. Safe motherhood program depicts the improvement like ANC visits (3 or more), information provided for delivery care and JSY program in village shows significantly higher proportion of institutional delivery compared to their counterparts in all the states.

**Table 3:** Percent institutional delivery in presence of program variables in EAG states.

Program variables	Uttaranchal	Rajasthan	UP	Bihar	Jharkhand	Orissa	Chhattisgarh	MP
**Delivery program**								
**ANC visit**								
No or <3	15.6***	33.0***	17.8***	22.5	7.1***	24.7***	7.1***	32.2
3 & more	52.0***	65.9***	37.8***	42.8	28.7***	53.4***	18.8***	59.3***
**Information on institutional delivery**								
No	16.1***	29.5***	13.2***	17.6	8.1***	18.2***	6.0***	25.3***
Yes	29.7***	43.1***	25.7***	34.6	17.4***	42.4***	14.4***	44.0***
**JSY program in village** ^@^								
No	22.3*	45.4*	19.4***	24.9	12.1***	31.7***	9.0***	31.1***
Yes	28.6*	40.4*	23.5***	28.8	14.0***	45.3***	13.7***	40.9***
**Accessibility and proximity to health services:**								
**Nearest public Health center providing delivery care**								
<10km	26.9	44.9***	23.0***	29.7***	15.3***	40.7***	14.7***	41.8***
10 km & above	22.1	31.4***	16.6***	22.2***	11.4***	31.3***	9.2***	36.6***
**Accessible public HC throughout the year**								
No	10.3***	33.7*	15.2***	21.4***	10.7*	33.1*	11.1	34.8**
Yes	29.0***	40.8*	22.1***	30.0***	13.3*	40.3*	13	40.9**
**Accessible private HC throughout the year**								
No	13.6***	36	14.9***	21.5***	12	30.8**	10.7	35.2**
Yes	28.6***	40.8	22.2***	29.9***	13.2	40.6**	13.2	41.0**
**Program under state program to encourage service utilization:**								
**ASHA in village**								
No	26.7	39.1	20.7	26.5	13.2	36.6	12.4	38.3
Yes	25.7	42.1	23.2	29.1	12.6	43.3	13.2	41.8
**ANM residing in village**								
Outside & >5 km	27.2	43.5***	21.0**	27.3	11.9*	43.8*	12.9	41.7*
residing <5 km	26.1	39.3***	22.4**	27.8	13.5*	38.9*	12.5	38.3*
**Skilled ANM**								
No	26	39.8***	21.4**	26.8***	13.1	38.3***	12.2	39.2*
Yes	27.3	45.2***	23.1**	29.3***	13.1	53.3***	14.1	41.8*
**Doctor available at PHC**								
No	20.1*	37.1***	21.2	24.3*	5.7***	45.1***	12.1	39.5
Yes	28.0*	42.5***	21.9	27.8*	13.4***	38.1***	13	39.6
**Manpower adequacy at PHC**								
<60% (3rd quintile)	28.2**	39.1***	22.1	28.2	10.9*	45.1***	12.3*	38.0***
>60% (3rd quintile)	14.4**	44.0***	21.3	27.1	13.3*	36.2***	14.2*	42.7***
**Drugs adequacy at PHC**								
<60% (3rd quintile)	24.4	40.1	21.3	29.7	16.1**	34.1***	12.2	37.3
>60% (3rd quintile)	26.8	40.8	22	26.8	12.4**	51.7***	12.8	42.3
**Physical infrastructure adequacy at PHC**								
<60% (3rd quintile)	35.3	36.1***	22.1	31.1	11.5*	42.7***	12.4	42.1
>60% (3rd quintile)	25	41.1***	22	27.6	13.9*	53.1***	13.8	41.3
**Essential instruments & laboratory services adequacy at PHC**								
<60% (3rd quintile)	26.2	40	21.8	27.2**	12.6*	39.7	13	37.6***
>60% (3rd quintile)	29	45.5	21.7	33.0**	13.5*	40.7	11.6	42.3***
**Infrastructure adequacy at HSC**								
<60% (3rd quintile)	21.9**	42.3*	21.7	27.3***	12.6*	38.1***	10.5*	39.2
>60% (3rd quintile)	28.4**	40.0*	21.9	36.7***	14.1*	44.8***	13.2*	40
**Number of deliveries at HSC & PHC in district**								
Less than mean	26.4	39.9***	22.1*	26.3*	13	39.5	12.4*	40
More than mean	26.4	46.0***	20.0*	28.2*	13.5	41.8	16.2*	39.1
**Total (%)**	25.4	39.9	20.4	26.4	12.4	38	11.7	38
**Total (N)**	1017	7094	16035	9492	8587	4200	3281	5337

Tests of difference (t-test) by program variables

*p<0.05,

**p<0.01,

***p<0.001

^@^: based on JSY beneficiary in last one year

Accessibility to the health centre definitely played a very important role in the utilization of services and was assumed to have a negative association. The respondents prioritized the accessibility of public health centre vis-à-vis that of a private health center, since the respondents could access beneficial rural health programs through public health centers. Nearest health center significantly shows higher proportion of delivery in all states except for Uttaranchal. This study showed that the propensity for delivery care significantly decreased as the distance from women’s residence to a public health centre increased; chances of an institutional delivery reduced drastically in health centers which were located at a distance of 30 kilometers or more in all the states as compared with births to women living within five kilo-meters of a hospital. Minimum utilization was observed in Jharkhand and Chhattisgarh followed by Uttaranchal and UP. The role of a skilled ANM was more important for utilization of services in Rajasthan, Bihar, Orissa and MP. Availability of basic infrastructure required for delivery care at health center shows variation in preferences for institutional delivery by states for instance availability of doctor at PHC shows significantly greater probability of delivery care in most of the EAG states except in UP and Chhattisgarh while other health personnel at PHC have not created much difference on utilization; similarly drugs adequacy and availability of physical infrastructure does not show significant variations in states for utilization except for Orissa and MP. Adequate laboratory services/equipment required for delivery care has shown significantly an increased utilization in improving states of Madhya Pradesh, Orissa and Rajasthan including in Uttaranchal and Bihar.

Satisfactory, adequate physical infrastructure at HSC attracted more women for utilization mainly in Uttaranchal, Bihar and Orissa. The study demonstrated maximum utilization and delivery at public health centre (HSC/PHC/CHC) in similar states. The reason could be the fact that many rural health centers were poorly staffed, offered a limited range of services, and typically lacked the special equipment, supplies, and medicine needed to provide delivery care. These findings are similar to a study conducted by PAHO [31], which found that predisposing, enabling and accessibility factors are stronger predictors of delivery care utilization than some of the health programs and infrastructure present in health centers. The results from correlation matrix showed insignificantly weaker association of ANC with accessibility to health center (0.007) and with percent urban (0.006); similarly for household wealth quintile with improvement in the public health center (-0.001).

### Multilevel logit results

This study used multilevel model in order to see the effect on utilization due to village and due to district level variations. Model-1 included characteristics of the mother (or women inter-changeable since one child corresponds to one woman, women are unit of analysis and so women’s characteristics are considered.) assumed as predisposing, enabling and need factors; model-2 assumed environmental and community factors as program variables and model-3 (full model) included all the three level variables in the multilevel analysis. Since these EAG states are categorized with similar social-cultural and programmatic characteristics hence state wise modeling in the analysis is avoided and differentials due to village/district level are explored to strengthen the decentralized program in efficient way.

#### Model 1 (predisposing, enabling and need factors)

Women socio-economic and individual characteristics were controlled in model-1 to see their marginal effect. Increasing age at births increased the utilization of health institutions for birth since increasing age at birth could magnify the risk of complications during delivery. Women belonging to non-SC/ST castes had a higher probability of institutional utilization while increasing birth order and child care burden decreased the likelihood of having institutional births. Surprisingly, working women were significantly less likely to utilize institutional health facilities, owing to the fact that their engagement in agricultural labor and work pressures did not permit them to access institutional health facilities for deliveries.

Undoubtedly, increasing educational levels, existence of government awareness programs around delivery care (odds = 1.7), women belong to upper wealth quintiles (odds ranges = 1.3–3.6) and had at least 3 ANC (odds = 2.4) incredibly more likelihood of institutional birth. Problems during delivery and earlier pregnancy loss increased the odds (odds = 1.6) for utilization which are their perceived belief and requirement to go to the health institution. The study found variation in service utilization ‘between districts’ and ‘between villages’ to be 10 percent and 12 percent respectively.

#### Village-level effect model 2 (program variables)

Village level included external environment and community health program variables (Table 4). Here external environment refers to the accessibility and availability of the health center. The State health program (NRHM) requires the involvement of health personnel in community, and the availability of functional infrastructure in the public health center. Distance was a major factor determining the utilization of services, as this model demonstrates adequately. Utilization was inversely proportional to the distance from institutional health facility: this study showed a rapid decline of more than 50 percent in the chances of having an institutional delivery once the distance to public health centre increased by 10 km or more. Additionally, skilled ANM in village, functional PHCs and observed improvement in HSC/PHC/CHC (in last one year) had positive influence and significantly more probability of utilization as compared to non-skilled, non-functional PHC and not-good improved health center (p<0.1).

**Table 4:** Three level model: Multilevel weighted logistic regression estimates for institutional births, EAG states, 2007–2008.

Selected variables	Model 1		Model 2		Model 3	
	odds	CI	odds	CI	odds	CI
**Enabling factors**						
**Information on institutional delivery**						
No^@^						
Yes	1.71***	(1.79–1.63)			1.70***	(1.78–1.62)
**3 and more ANC**						
No^@^						
Yes	2.43***	(2.49–2.37)			2.43***	(2.49–2.36)
**Wealth quintile**						
Poorest^@^						
Second	1.31***	(1.39–1.23)			1.30***	(1.38–1.22)
Middle	1.57***	(1.66–1.48)			1.53***	(1.62–1.43)
Fourth	2.08***	(2.19–1.97)			2.00***	(2.11–1.89)
Richest	3.59***	(3.74–3.44)			3.37***	(3.52–3.22)
**% household with higher education**					74.59***	(75.0–74.16)
**% urban by district**					2.20***	(3.08–1.32)
**% poorest household by district**					0.41***	(1.08-(-0.268))
**Need factors**						
**Pregnancy loss in last 5 years**						
No^@^						
Yes	1.57***	(1.68–1.45)			1.55***	(1.67–1.43)
**Problem during delivery**						
No^@^						
Yes	1.60***	(1.67–1.53)			1.61***	(1.68–1.54)
**Environmental factors**						
**External environment factors**						
**Public health accessible**						
No^@^						
Yes			1.02	(1.17–0.87)	1.04	(1.23–0.84)
**Private health center accessible**						
No^@^						
Yes			1.09	(1.25–0.93)	1.08	(1.26–8.89)
**Distance to public health center providing delivery care**						
<10 km^@^						
10-30km			0.69***	(0.74–0.64)	0.86***	(0.94–0.77)
30+ km			0.52***	0.64–0.40)	0.68***	(0.98–0.38)
**Community health program variables**						
**ANM in village (<5km)**						
No^@^						
Yes			1.01	(1.07–0.95)	1	(1.07–0.92)
**Skilled ANM (skill attendant)**						
No^@^						
Yes			1.12**	(1.17–1.07)	1.07*	(1.15–0.99)
**Functional PHC**						
Yes^@^						
No			0.90***	(0.96–0.84)	1.03*	(1.12–0.94)
**Improved public health facility (SC/PHC/CHC)**						
Not good^@^						
Good			1.16***	(1.23–1.09)	1.02	(1.09–0.94)
Very good			1.21***	(1.35–1.07)	1.10*	(1.26–0.94)
**Manpower adequacy at PHC**						
Less than 60% ^@^						
More than 60%			1	(1.05–0.95)	1	(1.09–0.90)
**Drug adequacy at PHC**						
Less than 60% ^@^						
More than 60%			0.85***	(0.92–0.79)	0.89	(0.98–0.79)
**Equipment/lab services adequacy at PHC**						
Less than 60% ^@^						
More than 60%			1.24***	(1.32–1.16)	1.09**	(1-19-0.98)
**Infrastructure adequacy at HSC**						
Less than 60% ^@^						
More than 60%			1.14	(1.21–1.07)	0.94	(1.02–0.85)
**Average number of delivery at SC/PHC by district (log)**			1.18***	(1.22–1.14)	1.31**	(1.35–1.26)
**Fixed Part**						
**Cons**	0.047***	(0.047-.047)	0.209***	(0.39–0.04)	0.054***	(0.47-(-0.362))
**Intra-class correlation (variance partition coefficient)**						
**Between district**	0.105		0.153		0.142	
**Between PSU**	0.127		0.108		0.107	
**-2** * **log likelihood**	**13076.6**		**46837.4**		**12681**	

Controlled for other predisposing and enabling factors

^@^ Reference category

SE: standard error

*p<0.1,

**p<0.05,

***p<0.01

Adequacy of infrastructure had a different impact on the utilization. Adequacy (upper 3^rd^ quintile) of essential equipment/laboratory services required for maternal care at the PHC and adequate physical infrastructure (including manpower, drugs and equipment recorded in DLHS data) at the HSC increased chances of services utilization by 24 percent and 14 percent respectively as compared to poor adequacy of facilities. Increased average number of deliveries at PHC and HSC implies an increasing likelihood of institutional delivery, which could be a good indication of improving rural health programs. These results support the study by Stephenson and Tsui (2002) who found that in Uttar Pradesh, presence of secondary health facility considerably enhanced care-seeking for both pregnancy and childbirth.

#### Full model (model) 3

Full model included the variables at all the three levels; similar results were obtained as in model 1 and 2, except for only some changes in the values of odds in analogous manner. District variables like percent of relative neighborhood poverty, percent of neighborhood higher education and percent urban have significant influence on the utilization. A significant 74 percent increase in the utilization by increasing one percent change in higher educated population in village, increase if women belong to more urbanized district and less utilization if women belongs to poorest (lowest WQ) district. Inter district variation was found to be 14 percent whereas 11 percent variation was observed due to village at level 2 in utilization once controls for all the three level variables in the model.

**Fig 3:**
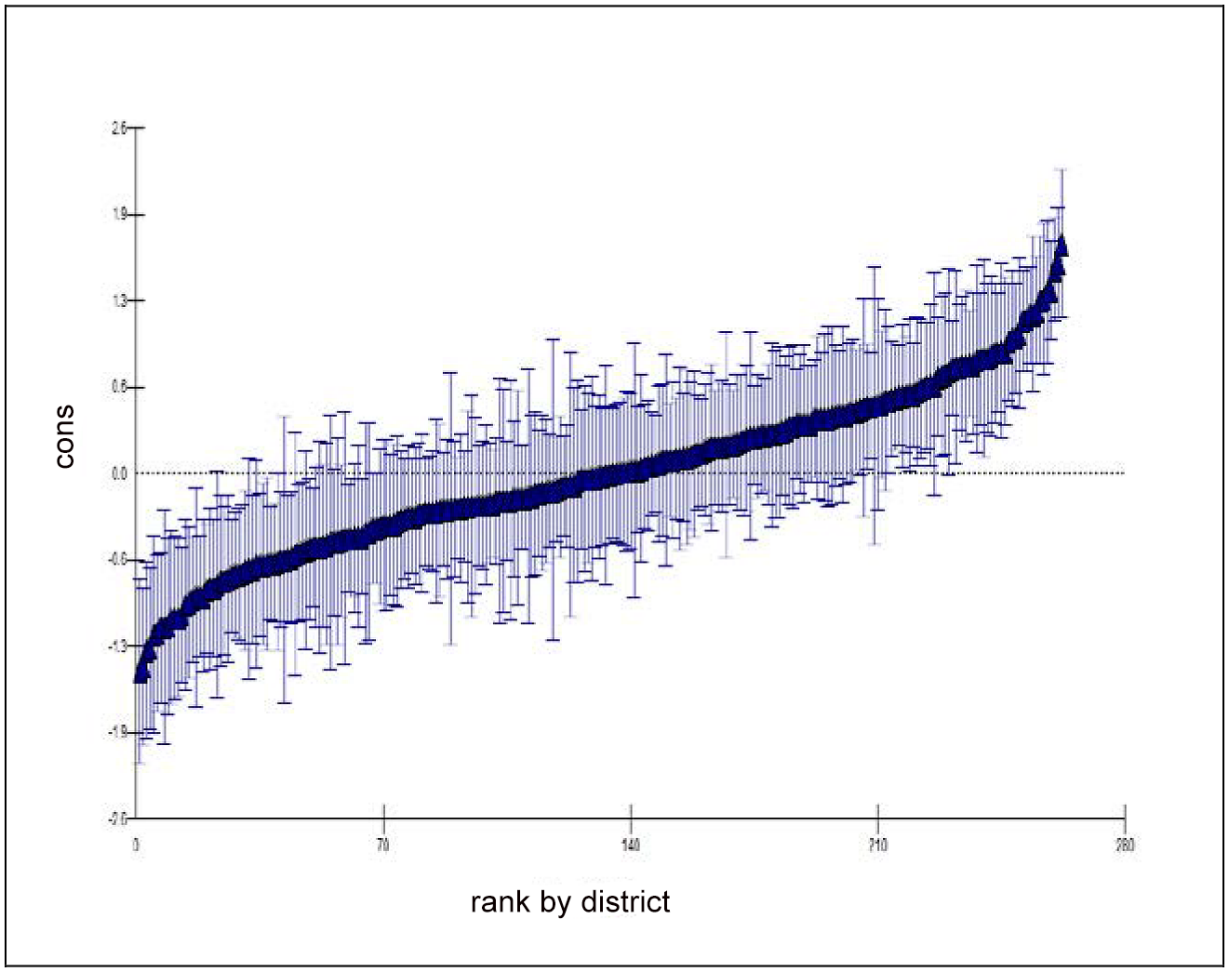
Residual plot for the district of EAG states (Multilevel-Logit Model). Residuals of each district (n = 263) are ranked and plotted for the outcome variable institutional delivery with 95% confidence interval.

**Fig 4:**
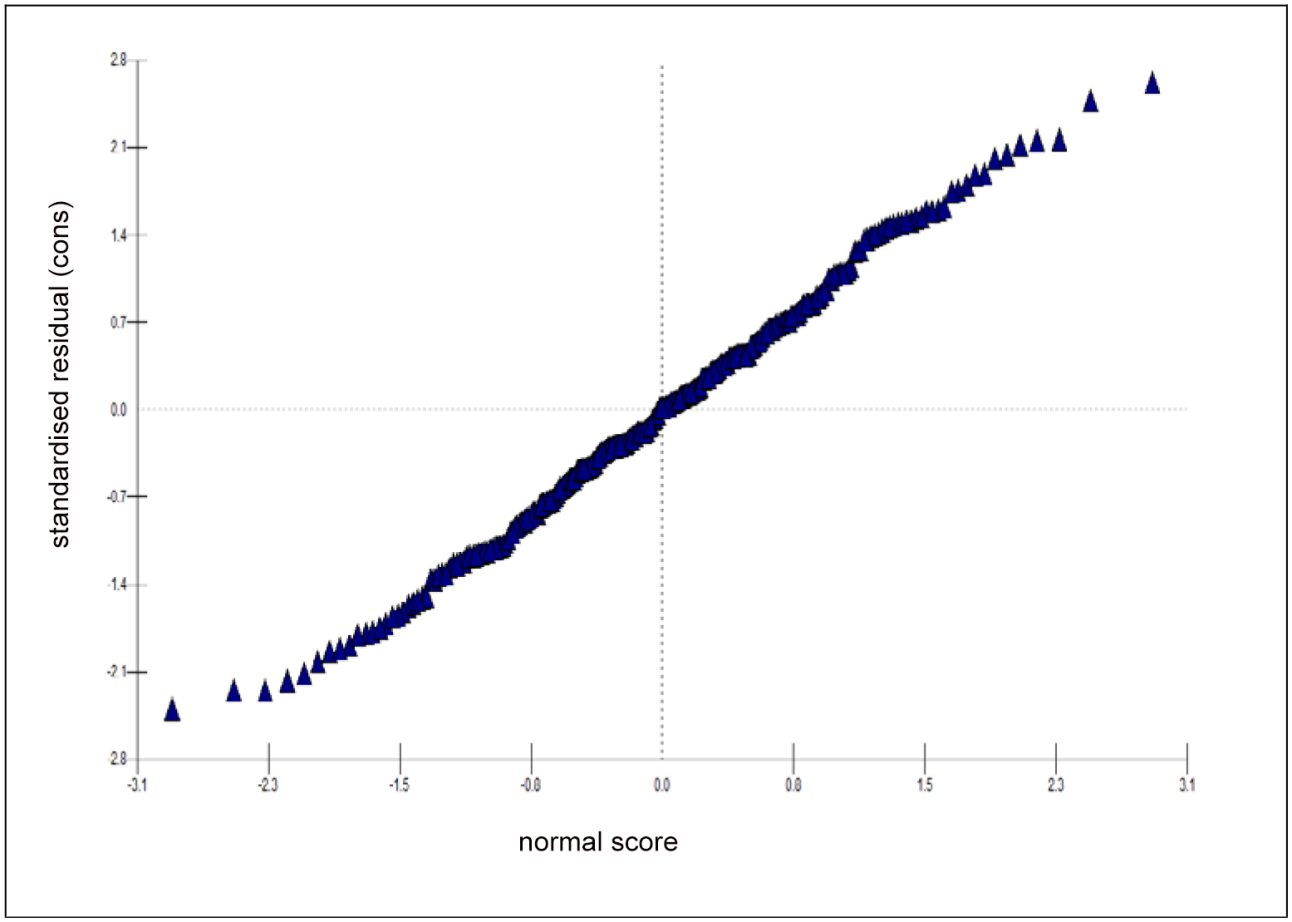
Normal plot for standardized residuals and normal scores for the district of EAG states. Since the residuals for outcome variable (institutional delivery) are normally distributed hence this regression model explains all trends in the random dataset.

Fig 3 shows the full model residual map (three-level logit-model) with the confidence interval range (at 5% level of significance) and Fig 4 shows the normal probability plot for the outcome variable institutional delivery by districts of EAG states.

## Conclusions

The main purpose of this study was to examine the variation and pattern of utilization services for institutional births by states in existence of external or environmental variables. Utilization by states demonstrated that there has been an improvement in utilization of services in the states of Rajasthan, Orissa and MP; importantly, there was also a satisfactory prevalence of utilization found in Bihar and Uttaranchal. States of similar social and cultural settings are found to be dissimilar improvement in maternal care in presence of same safe motherhood program (NRHM, 2005) launched at same time. Test of difference shows the significant variation by program variables between states. Undoubtedly, Safe motherhood program illustrates the improvement such that regular ANC (3 or more visits), information provided for delivery care and JSY program in village shows significantly higher proportion of institutional delivery compared to their counterparts in all the states. Nearest health center significantly shows higher proportion of delivery in all states except for Uttaranchal. Availability of basic infrastructure required for delivery care at health center shows variation in preferences for institutional delivery by states; for instance availability of doctor at PHC shows greater probability of delivery care in most of the EAG states except in UP and Chhattisgarh while study shows that adequate laboratory services/equipment required for delivery care encouraged utilization in improving states of Madhya Pradesh, Orissa and Rajasthan including Uttaranchal and Bihar.

This study used a three—level multilevel model to study the effect on utilization due to village and district level variations controlling with predisposing, enabling and health program factors together. Remarkably, increasing age at births increased the likelihood to have institutional utilization, while increasing birth order and child care burden decreased the likelihood of institutional births. Enabling factors higher level of education, involvement of maternal health care program showed more likelihood for institutional birth. Birth risk like complications during delivery and earlier pregnancy loss increased the chance of utilizing hospital. Results confirm that inaccessible and non-availability of health center was major hindrances for service utilization. Utilization rapidly declined by half as the distance to health center increased by 10km or more. Additionally grass-root level inclusion of health personnel involvement in community and infrastructure available to public health center like skilled ANM in village, functional PHC and observed improvement in public health facility in last one year contributed briefly to more probability of utilization. Adequacy of infrastructure showed a different pattern vis-a-vis utilization.

Drugs and manpower adequacy could not influence the utilization however, adequacy of essential equipment/laboratory services required for maternal care at PHC and adequate infrastructure at HSC has shown to increase the chances of services utilization significantly as compared to facilities with poor adequacy. Increasing number of deliveries at primary health centers implied higher proportion of institutional births, which can be a good indicator of improving rural health programs. This need is to accelerate the utilization by improving the adequacy at facility level, early identification of high risk mothers, and infection control and management. Percent of relative neighborhood poverty, percent of neighborhood higher education and percent urban have great influence on the utilization. Neighborhood poverty was negatively related to utilization while community higher education and if women belonged to more urbanized districts has significantly increased the utilization. Partly study supports that indifference in the behavior of health care system functioning, fail to provide equity services to vulnerable subgroup of strata in terms of accessibility, variety and quality of services [32].

Study concludes that apart from socio-economic characteristics of women which hinder service utilization, it is the infrastructure adequacy and accessibility to services. Any attempt to increase maternal care-seeking behavior in rural India will require resources to be targeted at the most impoverished areas and development of strategies for reaching those not yet reached. Increasing the proportion of women cared-for in health facilities and by skilled health providers during pregnancy and childbirth is critically important for improving the health of mothers and new born babies. Community behavior reflects in utilization hence community based program should be encouraged. Evidence from this study suggests that the mere availability of health facilities is necessary but not sufficient condition to promote utilization, if the quality of service is inadequate and inaccessible considering the inter-districts variation for program implementation. However, some contradictory results on adequacy and delivery care has come up from this study. Infrastructure adequacy indicators of drugs, and manpower required at health center did not encourage utilization in some of the states. The reason could be unexplained factors for instance rigid cultural norms to deliver baby at home, less faith or less attraction on public health centers over private health center preference or others.

## Limitations of the Study

It was an opportunity to explore facility information to structure for insufficient study on the adequacy of health infrastructure availability and its linkages to utilization at district or state level in India though secondary data DLHS-3 had its own limitations. Study on facility adequacy restricted to public health facilities only. A more complete representation of determinants would have included perceived quality of obstetric services and pregnancy/delivery-specific care by women but such specific data were not collected in any survey data. Frequency of delivery at public health facility and private health facility could be examined from the hospital based data to monitor the quality of services provided by them. Rural-urban differential in institutional delivery and separate work on utilization of public-private health facility are some of the commonly identified areas to deal with. However, we were able to address some of these factors by using percent urban in our analysis. Though we admit that it is important to address those, we could not do much due to the unavailability of data on private health facility at state/district level. Further studies based on primary data must utilize information on such variables while explaining service utilization for delivery care and perceived quality at health facility in India.

## Supporting Information

S1 Appendix Appendix A. List of variables included in computation of infrastructure indices at PHC and HSC. (PDF)

S1 Fig Framework: Modified from Andersen and Newman’s Framework of Health Services Utilization, 1995. (TIF)
