# Supplementary material for: Do Physical Proximity and Availability of Adequate Infrastructure at Public Health Facility Increase Institutional Delivery? A Three Level Hierarchical Model Approach
Source: PLoS One. 2015 Dec 21;10(12):e0144352. doi: 10.1371/journal.pone.0144352 (PMC4686327; doi:10.1371/journal.pone.0144352)
Supplement: S1 Appendix — (PDF) [file pone.0144352.s001.pdf]

Appendix A

List of variables included in computation of infrastructure indices at PHC and HSC

Note: Availability of each item is coded as 1 and 0 otherwise.

Indices	Items	Number of items
**PHC**		
**Manpower index**	1. a medical officer,<br>2. a lady medical officer,<br>3. a staff nurse,<br>4. a pharmacist,<br>5. a lady health veteran or health assistant,<br>6. a laboratory technician,<br>7. an auxiliary nurse midwife(ANM)/female health worker and<br>8. an additional staff nurse	8
**Physical infrastructure index**	1. a proper building for PHC,<br>2. cleanliness of its premises, ward and OPD;<br>3. at least four beds for patients,<br>4. regular water and electricity supply,<br>5. functioning toilet,<br>6. availability of a boiler,<br>7. a functional labor room,<br>8. a functional OT<br>9. OT has Boyles apparatus and anesthetic medicine, and<br>10. communication facility	10
**Essential drug index**	1. anti-allergics and drugs used in anaphylaxis,<br>2. anti-hypertensive,<br>3. anti-diabetics,<br>4. anti-anginal,<br>5. anti-tubercular,<br>6. anti-leprosy,<br>7. anti-filarials,<br>8. anti-bacterials,<br>9. anti-helminthic,<br>10. anti-protozoal,<br>11. antidotes,<br>12. solutions correcting water and electrolyte imbalance and<br>13. essential obstetric care drugs	13
**Essential equipment/ Laboratory**	**Functional instruments**<br><br>1. an examination table	39

**services index**	2. a delivery table, \<br>3. an OT table<br>4. bed side screen<br>5. a footstep<br>6. a shadowless lamp light for OT/labor room,<br>7. a macintosh for labor & OT table,<br>8. an oxygen trolley with cylinder and flow meter,<br>9. an instrument trolley,<br>10. a sterilization instrument,<br>11. an instrument cabinet,<br>12. a blood/saline stand,<br>13. a stretcher on trolley,<br>14. a stool for patients,<br>15. a wheel chair,<br>16. Almirah/Cupboard with lock and key<br>17. a separate dustbin for biomedical waste<br><br>**Delivery equipment**<br><br>18. a normal delivery kit,<br>19. a IUD insertion kit<br>20. equipments for assisted vacuum delivery<br>21. equipment for assisted forceps delivery,<br>22. equipments for newborn care and neonatal resuscitation,<br>23. a standard surgical set (for minor procedures like episiotomy stitching),<br>24. equipments for manual vacuum aspiration,<br>25. a baby warmer/incubator<br><br>**Cold chain equipment**<br><br>26. Ice Lined Refregerator (ILR) large<br>27. ILR small,<br>28. a deep freezer large,<br>29. a deep freezer small<br>30. a cold box<br>31. a vaccine carrier<br><br>**Requirement of the lab**<br><br>32. chemical for Hb testing,<br>33. reagent strips for urine albumin and urine sugar analysis,<br>34. rapid plasma regain (RPR) test kit for syphills,<br>35. reagent for peripheral blood smear examination for MP,<br>36. residual chlorine in drinking water testing strips,<br>37. centrifuge,	

	38. a light microscope<br>39. a binocular microscope	
**HSC**		
**Physical infrastructure index***	**Manpower**<br><br>1. female/male health worker,<br>2. additional ANM (contractual),<br>3. Skilled Birth Attendant (SBA)<br>4. any other health personnel in the village<br><br>**Physical infrastructure (functional)**<br><br>5. regular water and electricity supply,<br>6. toilet<br>7. labor room<br>8. proper biomedical waste disposal<br>9. examination table,<br>10. labor table,<br>11. foot step and<br>12. a bedside screen<br><br>**Required instrument for primary care of pre and post natal care**<br><br>13. sterilizer,<br>14. auto disposable (AD) syringes,<br>15. hub cutter,<br>16. BP instrument,<br>17. stethoscope,<br>18. weighing machine for adult<br>19. weighing machine for infant<br>20. haemoglobinometer<br>21. foetoscope,<br>22. SIMS speculum,<br>23. IUD insertion kit and<br>24. a vaccine carrier<br><br>**Essential drugs**<br><br>25. drug-kit (A, B),<br>26. IFA tablets,<br>27. vitamin A solution,<br>28. ORS packet,<br>29. injection gentamycin,<br>30. injection magnesium sulphate,<br>31. capsule ampicillin,	35

	32. tablet metronidazole,<br>33. tablet misoprostol,<br>34. tablet,Primaquine and<br>35. tablet/syrup Paracetamol	
