# Supplementary figures and images for: Do Physical Proximity and Availability of Adequate Infrastructure at Public Health Facility Increase Institutional Delivery? A Three Level Hierarchical Model Approach
Source: PLoS One. 2015 Dec 21;10(12):e0144352. doi: 10.1371/journal.pone.0144352 (PMC4686327; doi:10.1371/journal.pone.0144352)

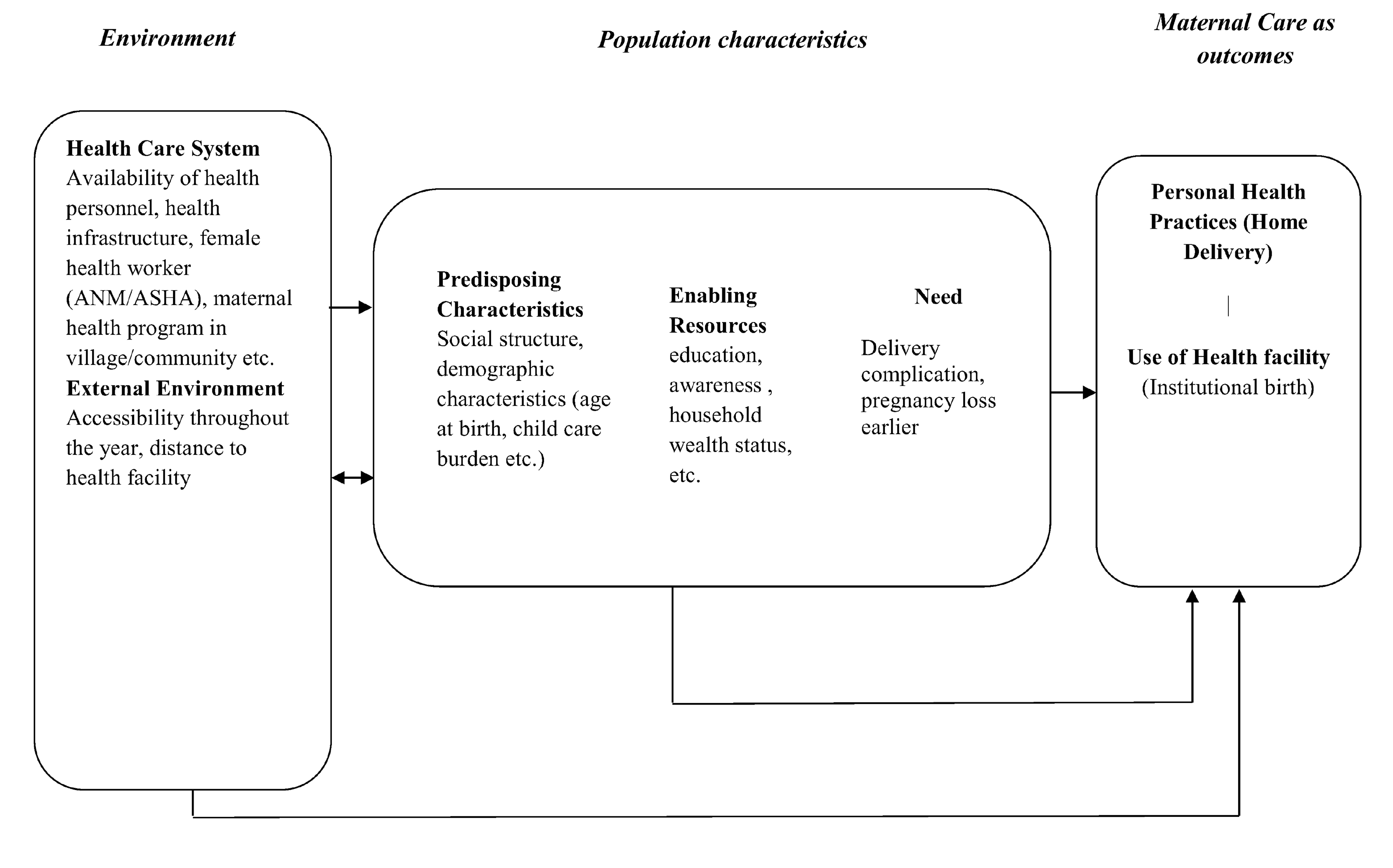

Supplement: S1 Fig — (TIF) [file pone.0144352.s002.tif]
